# Construct validity of a service-setting based measure to identify mental health problems in infancy

**DOI:** 10.1371/journal.pone.0214112

**Published:** 2019-03-28

**Authors:** Janni Ammitzbøll, Anne Mette Skovgaard, Bjørn E. Holstein, Anette Andersen, Svend Kreiner, Tine Nielsen

**Affiliations:** 1 National Institute of Public Health, University of Southern Denmark, Copenhagen, Denmark; 2 Department of Public Health, University of Copenhagen, Copenhagen, Denmark; 3 Department of Biostatistics, University of Copenhagen, Copenhagen, Denmark; 4 Department of Psychology, University of Copenhagen, Copenhagen, Denmark; IRCCS E. Medea, ITALY

## Abstract

Accumulating research document the needs of intervention towards mental health problems in early childhood. The general child health surveillance offers opportunities for early detection of mental health vulnerability, conditioned the availability of feasible and validated measures. The Copenhagen Infant Mental Health Questionnaire, CIMHQ, was developed to be feasible for community health nurses and comprehensive regarding the range of mental health problems seen in infancy. Previous testing of the CIMHQ has documented feasibility and face validity. The aim was to investigate the construct validity of the general population measure by using the Rasch measurement models, and to explore the differential functioning of the CIMHQ relative to a number of characteristics of the infants, local independence of items, and possible latent classes of infants. CIMHQ was tested in 2,973 infants from the general population, aged 9–10 months. The infants were assessed by community health nurses at home visits, in the period from March 2011 to December 2013. Rasch measurement models were used to investigate the construct validity of the CIMHQ. Analyses showed an overall construct valid scale of mental health problems, consisting of seven valid subscales of specific problems concerning eating, sleep, emotional reactions, attention, motor activity, communication, and language, respectively. The CIMHQ fitted a graphical loglinear Rasch model without differential item function. Analyses of local homogeneity identified two latent classes of infants. A simple model with almost no local dependency between items is proposed for infants with few problems, whereas a more complicated model characterizes infants with more problems. The measure CIMHQ differentiates between infants from the general population with few and more mental health problems, and between subgroups of problems that potentially can be targets of preventive intervention.

## Introduction

Children’s mental health problems are major challenges to public health [1], being the most frequent causes of learning disabilities and social and emotional impairment in childhood [1, 2].

Extensive research documents a population prevalence of mental disorders in school-aged children around 15% [1], and available studies of preschool children show similar figures [3–5]. A high risk of continuity of mental health problems across developmental stages has been found, and developmental disorders with onset in early childhood tend to persist in older ages [2, 6–9].

The clinical presentations of mental health problems and psychopathology in the first three years of life are described in the Diagnostic Classification of Mental Health and Developmental Disorders of Infancy and Early Childhood, DC:0-3R [10], in the ICD-10 [11], and in the Diagnostic and Statistical Manual of Mental Disorders (DSM-IV/DSM-V) [12, 13]. Population-based studies from recent decades have documented the existence of mental health problems and relational problems that impair young children’s development, emotional functioning and social relations [3, 4, 12, 14]; and that these problems can be reliably identified down to the age of 1½ years [15]. Among these, ICD-10 disorders of psychological development have been shown to affect 3% of the child population, whereas disorders of feeding, eating and sleep, and DC:0–3 disorders of emotional expression and regulation affect a total of 14% of 1½ years old children from the general population [16, 17].

Prospective studies have shown that infancy problems of development, and emotional and relational problems, predict mental health problems in later preschool and school age [18–25]. Also, infants’ problems of feeding and eating, and of sleep and emotional regulation are associated with an increased risk of emotional and behavioral problems later in preschool to school age [26–29].

Together, all available evidence points to the high impact, and long-term consequences of early mental health problems, underscoring the needs of intervention towards mental health problems in early ages [6, 30]. Unfortunately, only a minority of young children suffering from mental health problems are detected and referred to treatment [1, 31], with higher risk of unmet need the younger the age of the child. In line with this gap in intervention towards mental health problems in early childhood, the American Academy of Pediatrics has recommended regular screening of young children’s mental health and development, and the importance of validated instruments for screening in community settings have been stressed [30].

Still, major challenges remain to ensure the valid identification of mental health problems in community settings. Most important, there is a lack of comprehensive measures that capture the range and developmental presentations of mental health problems seen in the youngest children, and take into account the core issues of validity and feasibility in community settings as well [6, 30–34].

The Strengths and Difficulties Questionnaire (SDQ) [35] and the Child Behavior Check List (CBCL) [36] are well-established and well validated psychometric measures demonstrated feasible in general population of older children. Both measures cover the broad spectrum of mental health problems and psychopathology [37, 38, 39, 40]. However, none of these measures are available for use for children below the age of 1½ years [36, 41, 42].

Among measures published so far which are feasible for use in non-clinical populations of infants [37, 43, 38, 39, 44, 45] the following have been validated in community samples: 1) *The Ages and Stages Questionnaire (ASQ)* which measures domains regarding communication, gross and fine motor and problem solving from the age of one month [46], 2) *The Ages and Stages Questionnaire*: *Social Emotional (ASQ-SE)* measuring domains regarding compliance and adaptive skills including physiological needs and self-regulation from the age of three months [47], 3) *The Parents Evaluation of Development*: *Developmental Milestones (PEDS-DM)* which measures domains regarding fine and gross motor, expressive and receptive language, self-help and social-emotional behavior from birth [48], and 4) *The Brief Infant-Toddler Social and Emotional Assessment (BITSEA)* which measures social-emotional problems and delays in competences in domains of internalizing and externalizing problems, dysregulation, self-help, social relatedness and maladaptation from the age of 12 months [49]. The three questionnaires are developed and validated in US, they are answered by parents, and for the *ASQ/ASQ-SE* also by day-cares [43]. However, none of these measures covers the full range of infancy mental health problems as described in the literature [3, 4, 10, 12, 14, 38, 44, 40], and to our knowledge, none of the measures has been tested for use among health professionals in the general child health surveillance [43].

Community screening of infants’ mental health may be provided as a part of the general child health surveillance, which in many European countries includes services delivered by health visitors [50]. Research based on these settings indicates potentials for mental health screening for socio-emotional and behavioral problems in 1-year-olds [51], and 2-year-olds [52], psychosocial problems in 2-year-olds [53], and neuro-developmental problems in 30 months children [54]. A general population-based Danish study embedded in the settings of community health nurses (CHNs) has identified several infancy markers of psychopathology in preschool ages [22, 28, 55]. Among the findings from this study, particular potentials of screening were found at ages 8–10 months, however, conditioned the availability of validated and feasible measures to screen and intervene within the existing service settings [16, 55].

The Copenhagen Infant Mental Health Questionnaire (CIMHQ) was created to fulfill the need of a comprehensive infant mental health assessment feasible for use in the service-settings of CHNs [56]. In Denmark, CHNs deliver on average five home visits between the child’s birth and age 10 months, and among them the highest predictive validity regarding mental health screening was found in the home visit scheduled to child age 8 to 10 months [16]. Also, the literature on developmental psychopathology in early childhood points to this age as optimal regarding the validity of identification of deviations in development, the differentiation between normal and abnormal regulation and infant behavior, and regarding the potential subsequent intervention to follow the identification of mental health vulnerabilities [16].

The CIMHQ was created on theoretical evidence on key aspects of mental health and development within the following areas: sleep, feeding and eating, expression and regulation of emotions, curiosity and interests, attention, motor activity, communication and interaction, and language. These areas are also included in the usual routines of CHNs’ assessments, however previously without any standardized measurements (56). In order to comply with the existing routines of CHNs, the CIMHQ was designed to be short and easy to administer, counting a total of 27 items with a short descriptive text and elaborated guidelines [56].

Initial validation studies of CIMHQ have demonstrated the face-validity as judged by CHNs, showing high acceptance among CHNs as well as parents, and feasibility within the existing routines at home visits [56].

To further explore the validity of the CIMHQ, it is a key issue to determine to what extent the items measure *what we think they measure*, that is, whether the construct of CIMHQ captures mental health problems and developmental psychopathology in very young children when assessing infants from the general population [57].

The main goal of the present study is to determine 1) whether the CIMHQ can be used as a single and overall indicator of mental health problems in infants, 2) whether it collects redundant information, and 3) whether it functions equally good for children with and without mental health problems [57]. Specifically, we aim to investigate the construct validity of the general population measure by using the Rasch Measurement models, and to explore the issue of differential functioning relative to a number of characteristics of the infants, the local independence of items, and the possible latent classes of infants.

## Material and methods

### Study design and procedure

The study was embedded in the general health surveillance of CHNs in 11 municipalities located around the city of Copenhagen, Denmark, which have a mixed urban and suburban population. The setting was the scheduled home visits delivered by CHNs, of which the home visit at age 8–10 months is the focus for the present study [56].

In all Danish municipalities, the CHNs receive information from midwives about all registered childbirths, and all families with a new-born child are offered free home visits by a CHN in the first year of the child’s life. More than 90% of infant families attend, and mostly the same CHN visits to the same family [58, 59]. The main goal of the CHNs is to promote child health, via advising the parents and intervene when needed [56].

In the municipalities in the study area, the CHNs have applied standardized recordings since 2002 [58]. The recordings are stored in a clinical database, the Child Health database [60]. Data include midwives’ information on pregnancy and birth, and the CHNs’ recordings of child health and development assessed at home visits, as well as information obtained from parents regarding the child’s development and daily functioning, and the parents’ health, the family situation, and socio-economic conditions of the family (for details see Skovgaard et al., 2005 [58]). In the present study, a total of 45 CHNs participated, with only a few being replaced during the study period because of retirement or chance of workplace. Prior to the study, the CHNs were trained in the use of the CIMHQ. Ad hoc training was provided as well. Compliance of the CHNs was optimized during the study period by ad hoc supervision and two joint seminars (for a further description, see Ammitzbøll et al. 2016 [56]).

Parents were given written information about the CHN’s use of CIMHQ, and they were informed that their participation was voluntary. The parents gave oral consent for participation at the visit, and the CHNs completed the questionnaire in cooperation with the parents at the end of the visit. The study protocol has been assessed by the Research Ethics Committee of the Capital Region of Denmark, and the committee has stated, February 2011, that according to Danish legislation, ethical approval is not needed. The Danish Data Protecting Agency approved the project as a sub-project in the notification of the Child Health Database, J.nr. 2015-57-0008 and registration number 16–1055.

### Sample

The study population was 3,263 infants who were consecutively enrolled for participation in the period from 1^st^ of March 2011 to 31^st^ December 2013. The children were enrolled as part of the home visit scheduled at age 8–10 months, which in the present study was set to 9–10 months. A total of 290 children were not eligible because of invalid identification (n = 10), or address (n = 39), or because of severe physical or developmental illness or handicap (n = 15); because of parents did not speak or understand Danish language (n = 34), the parents declined (n = 48), or because of practical reasons (n = 105), or other reasons (n = 39).

The final sample was 2,973 infants who were eligible for the CIMHQ at age 9–10 months. Infants born before week 36 were included while adjusting for the gestational age of the child.

### Data collection

The practical procedures at the home visits were overall unchanged compared to existing practice, apart from the CIMHQ assessment, which took place at the end of the visit. The CIMHQ was completed in accordance with the guidelines in the manual, which included references to developmental milestones and functions of mental health [56]. As in existing routines, the assessment of the child was based on the CHN’s observations, as well as information from the parents, overall in accordance with the National guidelines [59].

### Measurements

The initial version of CIMHQ consisted of 27 items which cover the following areas or domains of infants mental health: 1. Sleep regulation (items A, B, C), 2. eating (items D, E, F, G), 3. expression of emotions (items H, I, J, K), 4. curiosity (item L), 5. concentration, attention and distractibility (items M, N, O), 6. motor activity (item P, Q, R), 7. communication and interaction (items S, T, U, V, W, X, Y) and 8. language (items Z, a), Table 1 shows the items of the CIMHQ.

**Table 1:** The Copenhagen Infant Mental Health Questionnaire domains, items and descriptions of items.

Sleep regulation	Stable sleeping pattern (A)	The child has established a steady pattern for sleeping and being awake
	Falling asleep time (B)	The child falls asleep within one hour
	Interrupted sleep (C)	The child is able to sleep at least three consecutive hours
Eating	Appetite regulation (D)	The child indicates clearly when it is hungry or full
	Eats too little (E)	The child has to be pressured to eat enough
	Refusal to eat (F)	The child refuses food even though it has not eaten for a long time
	Vomiting without otherwise being ill (G)	The child vomits more than once a week
Expression of Emotions	Generally happy and satisfied (H)	The child is happy and satisfied more than 80% of its waking time
	Often irritable, fussy, dissatisfied (I)	The child has at least two episodes every day where it is irritable, fussy, dissatisfied
	Cries often (J)	The child cries more than one hour every day
	Emotionally blunted (K)	The child shows no happiness, has limited facial expression and seems sad more than 50% of its waking time
Curiosity and interest	Curiosity, exploring (L)	The child shows interest in its surroundings, examines its toys
Attention	Is able to focus (M)	The child watch something or listen for more than one minute
	Maintain concentration (N)	The child is able to examine toys for more than two minutes
	Easily distracted (O)	The child is distracted by sounds, lights, movements, even while playing and does not return to its original activity
Motor activity	Generally increased level of activity (P)	The child is characterized by a high level of activity restlessness
	Generally reduced level of activity (Q)	The child has a passive motoric, is mainly inactive
	Impulsiveness (R)	The child is unpredictably active, throws things suddenly
Communication and interaction	Eye contact (S)	The child is able to establish eye contact. The Visiting Nurse is not in doubt that the child sees her eyes
	Contact smile (T)	The child smiles to the Visiting Nurse when eye contact is made
	Proximity seeking (U)	The child seeks contact with smiling, chattering, touching or reaching out after its parents
	Mutual communication (V)	The child uses gestures, smiles and chatter with its parents for more than two communication loops (answer><reply)
	Joint attention (W)	The child pays attention to parents’ indications, checks and looks again
	Bodily contact (X)	The child shows interest in bodily contact by expression and gesture
	Selectivity (Y)	The child clearly prefers the familiar care-personnel
Language	Language understanding (Z)	The child reacts to gestures/and some words
	Verbal expression (a)	The child expresses itself with facial expressions, gestures, pointing, chatter in syllables

All items are answered with “yes” or “no”. The coding of the items: E, F, G, I, J, K, P and R were reversed before analyses, so that a value of 1 signified presence of a problem, and a value of 0 the non-presence of a problem. The total score was obtained by summing individual item scores, i.e. a higher score indicated more problems.

### The Child Health Database

CHNs in the municipalities of the study area collect data prospectively by using standardized electronic records as part of the routines at home visits. These data are stored in the Child Health Database (CHD), and in this study we included child gender, child age at assessment, gestational age, birth weight, mother’s age at childbirth and Apgar score (The Apgar score is a measure of the newborn’s condition regarding circulation, neuro-motor activity and respiration, evaluated by the mid-wife) [61].

### Statistical analyses

Descriptive statistics were obtained using the SPSS version 22. Descriptive statistics was used to examine the differences between participants and non-participants (statistical testing by chi^2^, *p*-value <0.05) and correlations between items.

### Construct validity

To investigate the construct validity of the CIMHQ we used the Rasch measurement model (RM) for dichotomous items [62]. The following requirements need to be fulfilled for a set of items (i.e. a scale) to fit the RM model. 1) Uni-dimensionality: The items of a scale should measure only one underlying construct/latent variable (i.e. in this study the construct of mental health problems). 2) Monotonicity: The probability of a high item score should increase with increasing values of the latent variable (i.e. the probability of affirming the items towards a problem being present is increasing with the score on the scale). 3) Local independency (no LD): The items of the scale must be conditionally independent given the latent variable (i.e. the affirmation of any one problem should depend only on the level on the scale and not the affirmation of any other problems/items). 4) Absence of differential item functioning (no DIF): The items should be conditionally independent of exogenous (i.e. background) variables given the latent variable (i.e. the items should function equally for subgroups of the population for example boys and girls). 5) Homogeneity: The rank order of the item difficulties (or item parameters) should be the same for all persons regardless of their level of the latent variable (i.e. the order of the problems according to how hard they are is the same for all infants regardless of their level on the scale, or the easiest problem to have is easiest for all infants). The first four requirements are shared by all item response theory (IRT) models, while the last requirement of homogeneity is specific to the RM [63, 64].

Fit to the RM provides ideal measurement with the scale in question within the specific frame of reference that the analyses were undertaken in (i.e. infants 9–10 months, by CHNs in a home visit setting) [64, 65, 66], in the sense that: 1) the raw score is a sufficient statistic for the estimated person parameter, 2) the reliability of the scale is optimal, 3) measurement by the scale is criterion-related construct valid in Rosenbaum’s definition [63, 67], and 4) measurement by the scale is specifically objective [62, 63]. Sufficiency of the CIMHQ score would mean that the raw score contained all the information required to estimate the infant’s level of mental health problems. Specific objectivity would mean that measurement of infant mental health problems by the scale would be valid and unbiased within the frame of reference of infants in a community health care setting. The RM is the only IRT model, which provides sufficiency of the raw score and specific objectivity [63, 65].

If fit to the Rasch model is rejected, close to optimal measurement can still be achieved if the deviations from the Rasch models consist only of uniform LD between items and/or uniform DIF. Uniform LD or DIF occurs when the strength of the dependence between items (in the case of LD) or items and exogenous variables (in the case of DIF) is the same at all levels of the latent variable. These particular deviations can be incorporated and adjusted for in a graphical loglinear Rasch model (GLLRM) [68, 69], which is basically a Rasch model that allows these specific deviations [68].

### Item analyses by RM and GLLRM

The statistical software DIGRAM 3.24.0 [70, 71] was used for item analyses, as the implementation of GLLRM in this package provides formal tests for uni-dimensionality and sufficiency, as well as analyses of DIF and LD, while adjusting for false discovery rate (FDR) due to multiple testing.

Item analyses were conducted first for specific areas of mental health problems (subscales), followed by analyses of the overall scale (the total CIMHQ). All analyses were conducted using the following overall strategy: First, fit of the item responses to the Rasch model (RM) was tested. When fit to the RM was rejected, we proceeded with graphical loglinear Rasch analyses to test whether item responses fitted a more complicated GLLRM with uniform LD and/or uniform DIF relative to gender, age and Apgar score of the infants.

The overall fit of the models was tested using Andersen’s conditional likelihood ratio test (CLR), as was DIF at an overall level [72]. The fit of individual items was tested by comparing the observed item-rest-score correlations with the expected item-rest-score correlations under the model (i.e. as expected for items fitting the unidimensional Rasch model) [73]. In the GLLRMs, the presence of uniform LD and DIF was tested with conditional tests of independence, and using partial Goodman-Kruskal gamma coefficients to measure the conditional association between item pairs (LD) or between items and exogenous variables (DIF) given the rest-scores [74]. A critical level of 0.05 was used for all tests, and the Benjamini-Hochberg procedure was applied to correct for FDR due to multiple testing, when appropriate [75].

In order to examine the potential presence of latent classes of qualitatively different groups of infants (i.e. groups of infants with the same score; score groups), we conducted a stepwise analysis of local homogeneity among infants with no or few problems and those with more problems [76]. The starting point of the analyses of local homogeneity was the most complex model, where item parameters are assumed to be different for each score group. Then, similarly to a backwards model search, we proceeded stepwise towards a simpler model. In each step adjacent score groups were compared pairwise, and a decision was made to collapse extreme score groups, if the item parameters in the group were equal based on conditional likelihood ratio tests. This process continued until the item parameters in the remaining score groups were equal.

## Results

Table 2 presents the characteristics of the CIMHQ study population (N = 3,253). No differences were found between participants (n = 2,973) and non-participants (n = 280), except for the peri-natal adversity index of Apgar score, with more non-participating infants having Apgar <10 (*p* = .01). Initial descriptive analyses showed that only one child scored problems at the item of curiosity and interest (L) and only two on the item of eye contact (S). Further, the generally reduced level of activity (Q) lacked correlation to other items. These three items were excluded from further analyses, leaving a total of 24-items.

**Table 2:** Characteristics of the study population (N = 3,253^1^).

	Participants % (n)	Non-participants % (n)	p-value^2^	Missing % (n)
Sex				
Boys	51.7 (1,538)	51.4 (144)		
Girls	48.3 (1,435)	48.6 (136)	.93	-
Gestational age				
< 37 weeks	5.3 (155)	5.6 (11)		
>=37 weeks	94.7 (2,773)	94.4 (187)	.87	3.9 (127)
Birth weight				
< 2500 grams	9.2 (211)	7.2 (14)		
>=2500 grams	90.8 (2,077)	92.8 (180)	.35	23.7 (771)
Mother’s age				
< 24 years	9.6 (251)	7.9 (19)		
>=24 years, <=40	86.8 (2,257)	90.0 (215)		
> 40 years	3.5 (92)	2.1 (5)	.32	12.7 (414)
Apgar score				
< 10 at 5 min.	17.6 (457)	24.4 (58)		
= 10 at 5 min.	82.4 (2,135)	75.6 (180)	.01	13.0 (423)

^1)^ N = 3,263, of which 10 were unidentifiable caused missing civil registration number.

^2)^ by Chi^2^ test.

### Item analyses

Each subscale of the CIMHQ as well as the 24-item total scale were analyzed by RM and GLLRM, which included testing for DIF relative to the gender, age and Apgar score of the infants. The analyses showed that of the seven subscales analyzed only the expression of emotions and language scales fit RMs, while the remaining five subscales each fitted GLLRMs, which were all adjusted for local dependence (LD) between two or more items (all p-values > .05). Accordingly, the analysis of the 24-item total scale fitted a GLLRM, adjusted for the many instances of local dependence as well. No evidence of DIF relative to the gender, age or Apgar scores of the infants was found in any of the analyses after correction for FDR (all p-values > .05), Table 3.

**Table 3:** Global tests of DIF and global tests of fit in resulting Rasch models (RM) or graphical loglinear Rasch models (GLLRM) for each subscale and the total scale of 24 items across domains.

Subscales/scale (Items)	Global test of DIF relative to									LD^2^ items in final model^4^	Global test of fit		
	Age			Sex			Apgar score						
	CLR^1^	*Df*	*P*	CLR^1^	*Df*	*P*	CLR^1^	*Df*	*P*		CLR^1^	*Df*	*P*
Sleep regulation (A,B,C)	7.2	6	.30	7.3	3	.06	7.4	3	.06	A-B	5.7	3	.13
Eating (D,E,F,G)	13.4	8	.10	6.3	4	.18	8.6	4	.07	E-F	6.5	4	.16
Expression of emotions (H,I,J,K)	2.3	6	.89	2.4	3	.50	1.8	3	.61	*None*	5.0	3	.17
Attention (M,N,O)	6.5	6	.37	4.3	3	.23	0.8	3	.84	M-N	0.1	3	.99
Motor activity (P,R)	2.7	2	.26	2.5	1	.11	0.1	1	.71	P-R	1.1	3	.77
Communication and interaction (T,U,V,W,X,Y)	10.2	12	.60	7.6	6	.27	2.4	6	.88	W-Y	7.0	6	.32
Language (Z,a)	1.1	2	.57	0.1	1	.72	1.2	1	.28	*None*	0.0	0	1.00
Total scale (All items)	103.8	78	.03^3^	56.3	39	.04^3^	14.3	39	1.00	A-B-C & D-E-F-G & H-I-R- T & J-K-P-O& M-N & X-U-Y & V-a	46.8	39	.18

^1^CLR = Conditional likelihood ratio test,

^2^LD = Local dependence,

^3^p above .05 after adjustment for FDR due to multiple testing using the Benjamini-Hochberg procedure.

**Fig 1:**
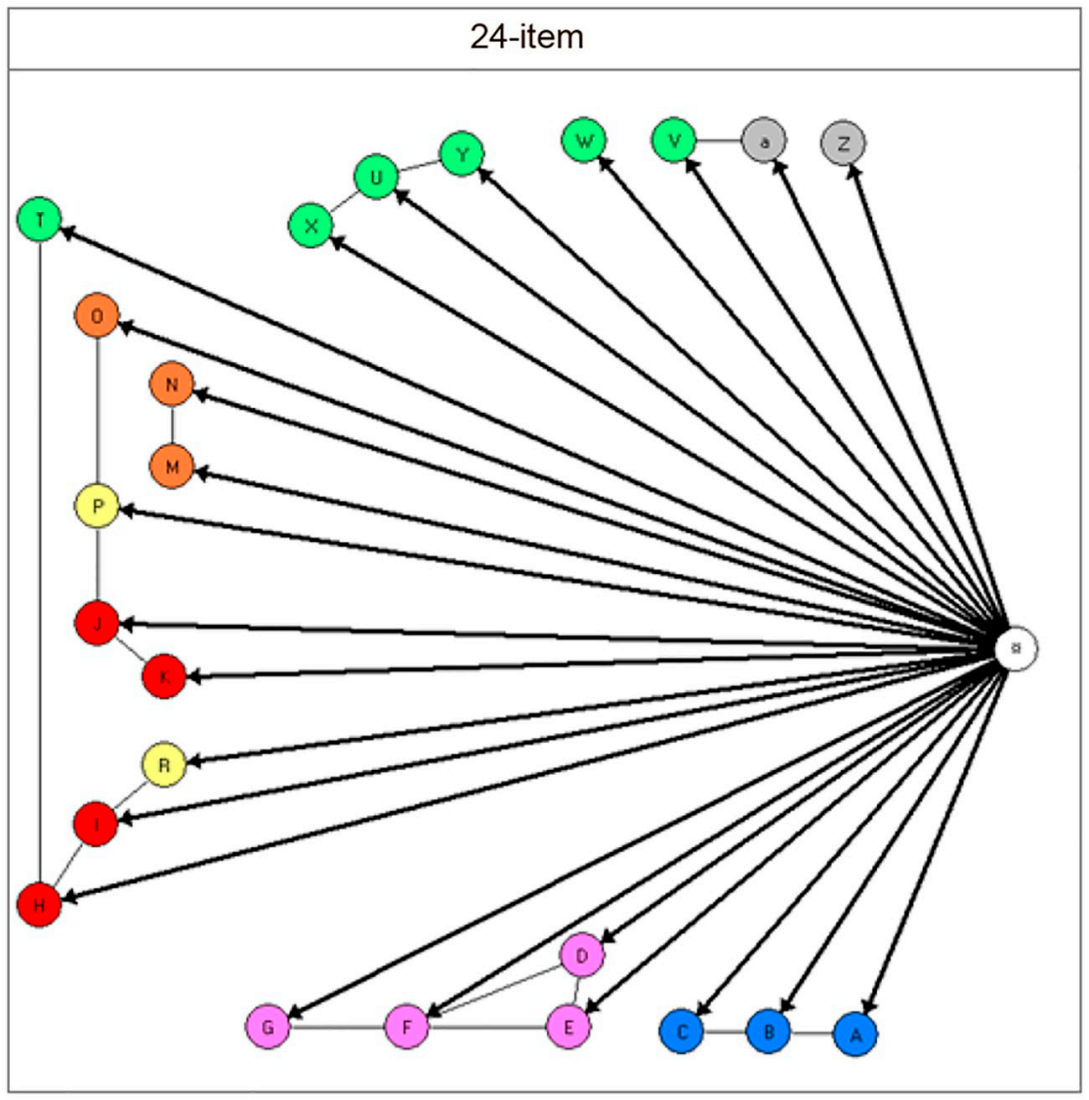
The graphical loglinear Rasch model for the 24-item scale. Note: The colour code signifies items from the seven different domains in CIMHQ: Blue: Sleep regulation (items A,B,C). Purple: Eating (items D,E,F,G). Red: Expression of emotions (items H,I,J,K). Orange: Concentration, attention and distractibility (items M,N,O). Yellow: Motor activity (items P,R). Green: Communication and interaction (items T,U,V,W,X,Y). Gray: Language (items Z,a). Connections between items signify that these items are locally dependent.

^4^The final model is illustrated in Fig 1.

The GLLRM for the 24-item CIMHQ scale for all infants included local dependence between many items—only item W (Joint attention) and item Z (Language understanding) did not violate the requirement of local independence. The locally dependent groups of items were: the three sleep items (A-B-C), the four eating items (D-E-F-G), two of the emotion items with one of the motor activity items and one of the communication and interaction items (H-I-R-T), two of the emotion items with one of the motor activity items and one of the concentration items (J-K-P-O), two of the concentration items (M-N), three of the communication items (X-U-Y), and finally one of the communication items and one of the language items (V-a) See Table 1 for item content, and Fig 1 for the final graphical loglinear Rasch model for the 24-item scale.

No problems were discovered with the fit of the individual items to the final subscales models and the 24-item total scale (all p-values > .05). Details of item fit statistics are given in Table A in the S1 File.

The analyses of local homogeneity identified two latent classes of infants with different item parameters (CLR 78.39, df 39, p < .0005), one consisting of infants with two or less problems (scores 0 to 2) and one of infants with three or more problems (scores 3 and higher). The frequency of infants with no or few (scores 0 to2) problems and more (scores 3 and higher) problems was 82.9% and 17.1% respectively.

**Fig 2:**
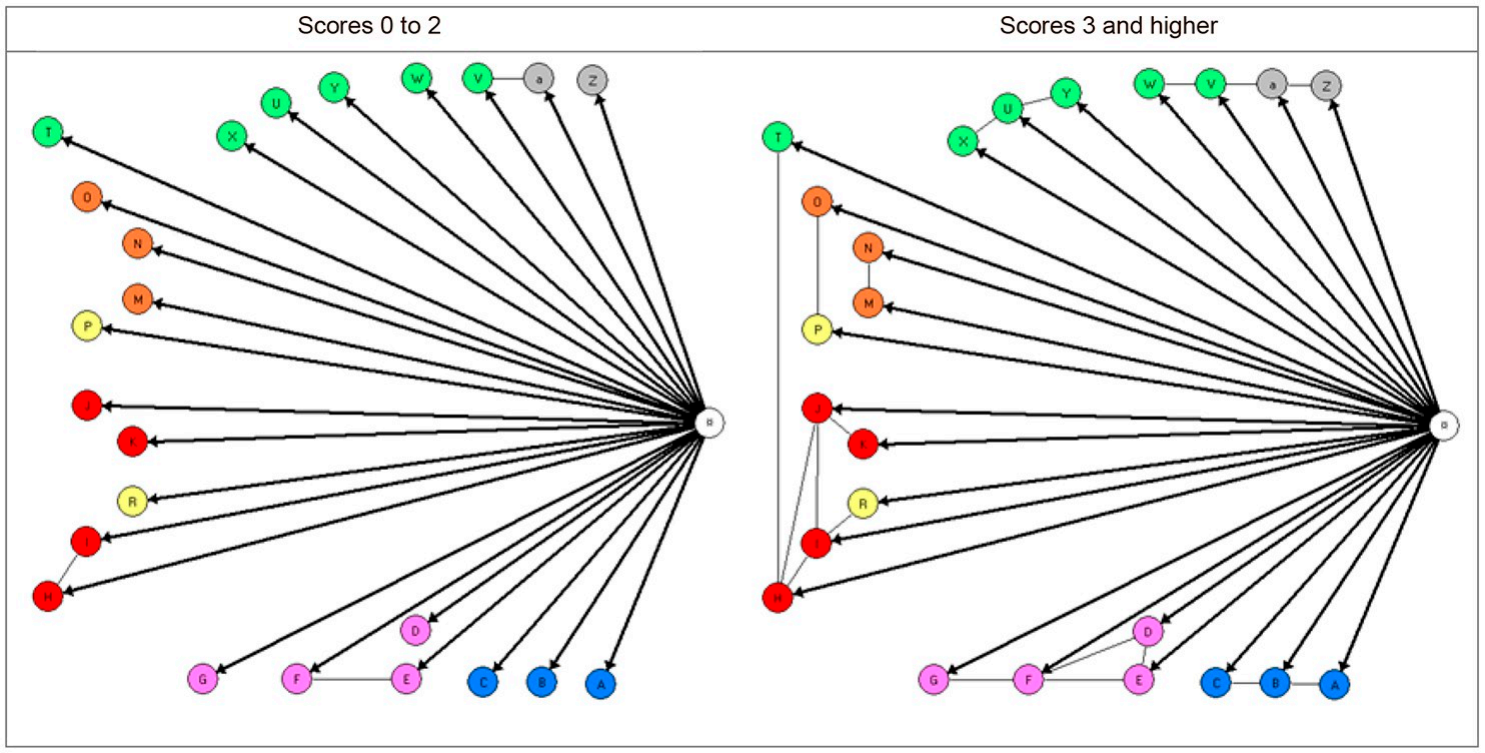
The graphical loglinear Rasch models for the two latent classes of infants scoring 0 to2 and 3 or higher, respectively, on the 24-item CIMHQ. Note: The color code signifies items from the seven different domains in CIMHQ: Blue: Sleep regulation (items A, B, C). Purple: Eating (items D, E, F, G). Red: Expression of emotions (items H,I, J, K). Orange: Concentration, attention and distractibility (items M, N, O). Yellow: Motor activity (items P, R). Green: Communication and interaction (items T, U, V, W, X, Y). Gray: Language (items Z, a). Connections between items signify that these items are locally dependent.

Subsequent analyses by graphical loglinear analyses of the two latent classes of infants resulted in two different graphical loglinear Rasch models (Fig 2). A simple model with only three pairs of locally dependent items was established for infants with scores of 0 to2 (CLR was 20.8, df 26, p = .75). Whereas, a complicated model with all items violating the assumption of local independence was established for infants with scores of 3 and higher (CLR was 61.0, df 42, p >.05 after adjustment for FDR), Table 4.

**Table 4:** Global tests of fit in graphical loglinear Rasch models for the two groups of infants defined by scores 0 to 2 and 3 and higher on the total CIMHQ.

Infants with scores	LD^1^ between items in GLLRM	Global test of fit		
		CLR^2^	*Df*	*P*
0 to 2	E-F & H-I & V-a	20.8	26	.75
3 and higher	A-B-C & D-E-F-G & H-I-J-K-R-T & M-N & O-P & X-Y-U & V-W-Z-a	61.01	42	.03^3^

^1^LD = Local dependence,

^2^CLR = Conditional likelihood ratio test,

^3^p above .05 after adjustment for FDR due to multiple testing using the Benjamini-Hochberg procedure.

For the class of infants with two or fewer problems the locally dependent item pairs were few. They consisted of two of the eating items (eats too little; refusal to eat), two of the emotion items (generally happy and satisfied; often irritable, fussy, dissatisfied), and one of the communication items and one of the language items (mutual communication; verbal expression) Fig 2.

For the class of infants with more problems the group-wise locally dependent items were: the three sleep items (A-B-C), the four eating items (D-E-F-G), the four emotion items with one of the motor activity items and one of the communication and interaction items (H-I-J-K-R-T), two of the concentration items (M-N), one of the concentration items and one of the motor activity items (O-P), three of the communication items (U-X-Y) and finally two of the communication and interaction items and the two language items (V-W-Z-a). See Table 4 for item content, and Fig 2 for the final graphical log linear Rasch models for the two groups infants separated by the score of 3 on the total 24-item CIMHQ. Details of item fit statistics are given in Table B in the S1 File.

## Discussion

We investigated the construct validity of the measure, CIMHQ, which has been created to help CHNs to identify infant mental health problems seen in the general child health surveillance, and to cover core areas of early developmental psychopathology [10, 12, 16, 56]. We used Rasch measurement models to assess the construct validity of the CIMHQ by exploring the issues of differential functioning relative to a number of characteristics of the infants, local independence of items and possible latent classes of infants. Our main findings are that CIMHQ identify two latent classes of infants, who are qualitatively different with regard to their numbers and patterns of problems, indicating that CIMHQ can be used as an overall scale to measure infancy mental health problems. Further, the seven subscales of sleep, eating, expression of emotions, concentration and attention, motor activity, communication and interaction, and language were found individually construct valid, indicating that they can be used separately to index early mental health vulnerability within specific areas [3, 4, 10, 14, 16, 77–79].

The locally dependent groups of items, which indicate problems within related areas (composite items), comprise the items on regulation of sleep (A-B-C), and the items on regulation of eating (D-E-F-G). These patterns of problems correspond to the symptoms of sleep disorders and feeding and eating disorders, respectively, as described in the age and developmentally appropriate diagnostic classification, DC:0-3R [10]. Among the groups of problems across areas, the composite items of the child’s negatively expressed emotions, unpredictable activity and interaction(H-I-R-T), the composite items of dysregulation of emotions, being easily distracted and increased level of activity (J-K-O-P) and the composite items of child being easily distracted (M-N) all correspond to the presentation of emotional and behavioral problems in infants and toddlers described in DC:0-3R [10]. The items of the child’s lack of selectivity, seeking bodily contact and proximity to familiar care-personnel (X-U-Y), and the items of mutual communication and language (V-a) concert with the complex symptoms of relationship disturbances and early symptoms of developmental disorders, as described in DC:0-3R [10, 22–24, 80, 81] and current diagnostic schemes of ICD-10 and DSM IV [11, 13]. Also, the two locally independent items joint attention (W) and language understanding (Z) correspond to symptoms seen in children with developmental disorders according to DC:0-3R as well as ICD-10 and DSM-IV [10, 11, 13].

We identified two latent classes of infants who were qualitatively different with regard to their number and patterns of problems. A simple model with almost no local dependency between items was proposed for infants with few problems, whereas a more complicated model characterized infants with more problems. In particular, complex patterns were seen between CHIMQ items of the child’s negatively expressed emotions, the child being easily distracted, impulsivity and increased level of activity. These findings may suggest possible early combinations of problems, which match the frequent co-occurrence of emotional, cognitive and behavioral symptoms seen in older children [3, 17], e.g. in mixed emotional and behavioral disorders, disorders of social functioning [82], and in disorders of hyperactivity [8]. In contrast, having problems of sleep, and eating occurred in patterns of problems from the same subscale.

Overall, the findings suggest that CIMHQ can differentiate quantitatively and qualitatively between infants with few or more problems, and the class of infants with three and more CIMHQ problems cut of 17.1% of the infants. These results are in line with the understanding of mental health problems in young children to span a continuum from normal developmental deviations to severe problems [78], and with the overall population prevalence of mental disorders seen in pre-school children [3, 4, 5]. The findings point toward a need of the CHN’s attention, and that the CIMHQ subscales could possibly guide an initial differentiated approach of intervention. However, the optimal threshold and combination of problems to guide intervention appropriately cannot be determined from the present findings [83, 84]. Therefore, more research is needed to explore the potentials of CIMHQ as an overall screener of single as well as complex problems of mental health vulnerability. Moreover, research in the possibilities as well as feasibility of intervention approaches based on CIMHQ is important to optimize the conditions of early mental health prevention in the settings of the general child health surveillance.

The major strengths of the study include the thorough investigation of construct validity in a large general population sample (n = 2973), of a comprehensive measure of infant mental health, which has shown its feasibility within existing service settings [56].

The use of IRT models to examine the construct validity of the new measure is a strength for a number of reasons [57, 69, 83, 85]: First, the Rasch analyses represent the current standards in measuring outcomes, providing detailed analyses of how items work within a scale. Second, the theoretically requirements are tested statistically. When establishing fit to a RM or a GLLRM without DIF, we know that the sum score is a sufficient statistic for the latent measure of mental health problems. As such the latent measure or the score can be used for assessment and comparative purposes as the score will not be confounded by any of the background factors included in the DIF analyses.

The study was embedded in general child health surveillance (CHS), which is the frame of assessment and surveillance of young children’s mental health in most Western countries [50]. So far, research data on validated measures for use in primary care has been very scarce [34, 43, 44]. The younger the age of the child, the less is known about measures to identify children in need of mental health intervention [86], and no other measures which investigate global mental health assessed by CHNs in children as young as the present population are available for comparison. Among validated measures for population-based identification of children at risk are the Strengths and Difficulties Questionnaire (SDQ) and the Child Behavior Check List (CBCL). Both cover a broad spectrum of mental health problems and psychopathology, and also account for the frequent co-existents of symptoms. However, they are developed for older children and have not been validated for use in children below the age of 18 months and none of them are suited to fit the agenda of CHNs working in a general child health surveillance [35, 36]. For comparison with CIMHQ, the CBCL is long and time consuming, and not feasible in existing settings of CHNs. SDQ is short, however not directly applicable in settings where the main agenda is guidance and interventions within the same setting, delivered by CHNs.

Among validated measures for population-based identification of infants at risk are the ASQ [46], the ASQ-SE [47], the PEDS-DM [48], and the BITSEA [49]. None of these measures are comprehensive with regard to the spectrum of mental health seen in young children, and in particular none of the measures include the common and important markers of mental health vulnerability expressed as problematic regulation of sleep and feeding and eating [29]. Further, these measures are answered by parents or day-care providers exclusively, and none of them provides detailed information on scale validity regarding current standards in measuring outcome, including co-occurrent mental health problems [57]. For comparison, the CIMHQ includes a broad range of problems, taking into account that mental health problems often co-exist across domains. The CIMHQ is answered by health professionals based on their assessment of the child, as well as information from the parents. All items are described in the manual and includes references to developmental milestones and functions of mental health to avoid inter-subjective variations of professionals when assessing a child [57]

Our study findings on children as young as 9–10 months illustrate that the CIMHQ captures early mental health problems well known to CHNs and the co-occurrence of problems which are relevant in the context of intervention [6, 16]. The pragmatic service-setting based approach is a strength of our study. Being based on existing routines of CHNs, the CIMHQ has potential to seek out infants, on whom the CHN has to take action already [54], but in a more standardized and validated way. This could possible guide a more differentiated apporach of intervention within the CHS in the municipalities. Hereby, the study findings add to the still very limited knowledge to guide interventions towards mental health problems in the earliest ages [40].

Some limitations need to be highlighted. First, non-participants (8.6% of the total population) could potentially lead to underestimation of infant mental health problems, due to higher frequencies of potential risk factors in non-participation children having severe physical and mental illness and handicap [4, 78], or families of low parental education and of not-Danish families as previously shown [56]. This issue should be a subject for future research. Second, other co-variates or exogenous variables than these included in the analyses could cause DIF, and future studies should further explore subgroups of the population. Third, there is a need for repeated studies in order to address the model uncertainty.

Nevertheless, the empirically data driven patterns of problems found in this study correspond to theoretical expected patterns of problems, indicating promising concordance.

## Conclusions

The infant mental health measure CIMHQ shows promising construct validity regarding identification of mental health problems in children from the general population. The findings suggest particular potentials for the detection of mentally vulnerable infants to guide intervention accordingly within the community child health surveillance. The validity of CIMHQ should be further tested in other samples and in other populations. Further research will how the scale can be used to guide intervention towards mental health problems in early childhood.

## Supporting information

S1 File PlosOne_Item fit statistics_CIMHQ.pdf. (PDF)
