# Supplementary material for: Construct validity of a service-setting based measure to identify mental health problems in infancy
Source: PLoS One. 2019 Mar 28;14(3):e0214112. doi: 10.1371/journal.pone.0214112 (PMC6438593; doi:10.1371/journal.pone.0214112)
Supplement: S1 File — (PDF) [file pone.0214112.s001.pdf]

Supplementary material

Table A. Item fit statistics in graphical loglinear Rasch models for the seven domain-specific subscales and the total 24-item CIMHQ.

Item	Domain subscales				24-item total scale			
	Observed gamma coefficients	Expected gamma coefficients	SD	*p*	Observed gamma coefficients	Expected gamma coefficients	SD	*p*
A	.84	.81	.038	.50	.52	.52	.046	.91
B	.80	.85	.039	.21	.39	.54	.064	.02[1]
C	.70	.70	.062	1.00	.43	.49	.053	.28
D	.72	.67	.046	.28	.55	.55	.041	.95
E	.83	.83	.023	.97	.58	.62	.030	.14
F	.84	.83	.023	.72	.61	.64	.030	.30
G	.52	.67	.059	.01[1]	.46	.50	.061	.46
H	.92	.87	.049	.35	.83	.73	.060	.11
I	.85	.85	.039	.92	.50	.47	.034	.42
J	.79	.86	.044	.15	.66	.52	.079	.08
K	.91	.89	.068	.72	.82	.59	.143	.11
M	.89	.90	.041	.81	.59	.54	.092	.53
N	.86	.85	.042	.88	.63	.46	.068	.02[1]
O	.79	.79	.050	1.00	.48	.45	.033	.36
P	.65	.65	.080	1.00	.62	.52	.045	.03[1]
R	.65	.56	.080	1.00	.37	.48	.053	.04[1]
T	.66	.72	.089	.48	.38	.48	.103	.35
U	.93	.75	.127	.17	.81	.68	.133	.31
V	.64	.69	.063	.34	.65	.57	.057	.17
W	.57	.57	.060	.91	.35	.41	.033	.07
X	.86	.74	.109	.25	.71	.49	.121	.08
Y	.40	.43	.104	.77	.34	.42	.063	.21
Z	.75	.75	.055	1.00	.46	.40	.054	.24
a	.75	.75	.055	1.00	.53	.49	.047	.41

[1] p above .05 after adjustment for false discovery rate (FDR) due to multiple testing by the Benjamini-Hochberg procedure.

Table B. Item fit statistics in graphical loglinear Rasch models for the two groups of infants separated by the score of 3 on the total 24-item CIMHQ.

Item	Infants with score 0-2				Infants with score 3+			
	Observed gamma coefficients	Expected gamma coefficients	SD	*p*	Observed gamma coefficients	Expected gamma coefficients	SD	*P*
A	.86	.85	.022	.93	.86	.85	.015	.61
B	.91	.92	.017	.61	.82	.84	.022	.35
C	.86	.87	.021	.68	.86	.84	.017	.27
D	.84	.84	.022	.73	.86	.86	.013	.71
E	.75	.75	.027	.87	.88	.88	.010	.52
F	.78	.79	.025	.83	.88	.88	.010	.99
G	.90	.90	.019	.82	.85	.84	.020	.71
H	.99	.99	.004	.76	.92	.87	.025	.05
I	.63	.64	.031	.80	.88	.88	.010	.56
J	.97	.97	.010	.90	.89	.86	.022	.21
K	.01	1.00	.003	.70	.89	.85	.041	.30
M	.98	.97	.010	.75	.83	.83	.029	.99
N	.97	.95	.014	.51	.83	.83	.019	.98
O	.58	.56	.033	.65	.87	.88	.010	.59
P	.89	.89	.019	.78	.84	.84	.013	.92
R	.84	.85	.022	.56	.85	.83	.018	.42
T	.96	.96	.013	.97	.87	.84	.035	.48
U	1.00	1.00	.003	.70	.88	.84	.046	.44
V	.95	.95	.012	.74	.85	.85	.018	.98
W	.44	.44	.035	.95	.86	.87	.011	.44
X	.99	1.00	.007	.94	.88	.82	.033	.78
Y	.87	.88	.020	.46	.82	.82	.020	.86
Z	.86	.84	.023	.52	.82	.84	.016	.31
a	.86	.86	.021	.77	.83	.85	.014	.23
